# Evolution of larval gregariousness is associated with host plant specialisation, but not host morphology, in Heliconiini butterflies

**DOI:** 10.1002/ece3.11002

**Published:** 2024-02-08

**Authors:** Callum F. McLellan, Stephen H. Montgomery

**Affiliations:** ^1^ School of Biological Sciences University of Bristol Bristol UK

**Keywords:** caterpillars, gregarious behaviour, Heliconiini, host defence, host plant, host specialisation

## Abstract

Insect herbivores, such as lepidopteran larvae, often have close evolutionary relationships with their host plants, with which they may be locked in an evolutionary arms race. Larval grouping behaviour may be one behavioural adaptation that improves host plant feeding, but aggregation also comes with costs, such as higher competition and limited resource access. Here, we use the Heliconiini butterfly tribe to explore the impact of host plant traits on the evolution of larval gregariousness. Heliconiini almost exclusively utilise species from the Passifloraceae as larval host plants. Passifloraceae display incredible diversity in leaf shape and a range of anti‐herbivore defences, suggesting they are responding to, and influencing, the evolution of Heliconiini larvae. By analysing larval social behaviour as both a binary (solitary or gregarious) and categorical (increasing larval group size) trait, we revisit the multiple origins of larval gregariousness across Heliconiini. We investigate whether host habitat, leaf defences and leaf size are important drivers of, or constraints on, larval gregariousness. Whereas our data do not reveal links between larval gregariousness and the host plant traits included in this study, we do find an interaction between host plant specialisation and larval behaviour, revealing gregarious larvae to be more likely to feed on a narrower range of host plant species than solitary larvae. We also find evidence that this increased specialisation typically precedes the evolutionary transition to gregarious behaviour. The comparatively greater host specialisation of gregarious larvae suggests that there are specific morphological and/or ecological features of their host plants that favour this behaviour.

## Graphical abstract

Heliconiini larvae are likely to be in an evolutionary arms race with their Passifloraceae host plants. By analysing larval social behaviour we investigate whether host habitat, leaf defences and leaf size are important drivers of, or constraints on, the evolution of larval gregariousness. Whereas our data do not reveal links between larval gregariousness and the host plant traits included in this study, we find that gregarious larvae are more likely to feed on a narrower range of host plant species than solitary larvae.

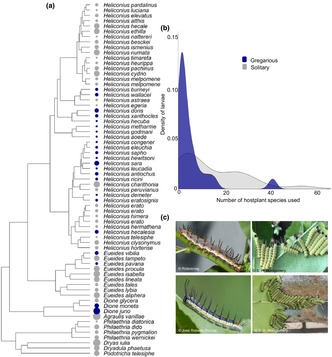

## 1 INTRODUCTION

Many prey animals have evolved grouping behaviour in response to predation and resource availability. Lepidopteran larvae benefit from aggregating in a number of ways, ranging from increased protection from predators (Greeney et al., 2012; Hunter, 2000; Reader & Hochuli, 2003) to facilitated feeding (Campbell & Stastny, 2015; Clark & Faeth, 1997; Fordyce, 2003; Kawasaki et al., 2009; Rentería et al., 2022). However, larval gregariousness also imposes costs, such as the facilitation of pathogenic infection spread and greater competition for food resources between siblings (e.g. Despland & Le Huu, 2007; Hochberg, 1991; Pescador‐Rubio, 2009), creating the context for possible evolutionary trade‐offs. Identifying key biotic and ecological factors that frame these trade‐offs may be critical for understanding the origin and evolution of gregarious behaviour. One of the most important of these ecological factors is larval host plants. As herbivores, lepidopteran larvae often have intimate evolutionary relationships with their hosts. These plants can act as a major source of selection for larvae, for example, due to their growth structure or by developing defences against herbivory that larvae must adapt to overcome (Birnbaum & Abbot, 2018; Clark & Faeth, 1997; de Castro et al., 2018; Despland, 2019; Karban, 2011; Thaler et al., 2002; Wittstock & Gershenzon, 2002).

Hostplant traits that may influence the evolution of larval gregariousness include their relative leaf size and anti‐herbivory defences. For example, larger leaves physically offer a wider surface area upon which larvae can collectively feed, which could be important if larvae benefit by remaining close to their group members, such as by reducing predation and parasitism risks (e.g. McClure & Despland, 2011). Across both short‐ and long‐term scales, plants are rarely passive in their evolutionary relationships with larval herbivores, having evolved a variety of defences in response to being selected as hosts. Evolutionary adaptations such as tougher leaf surfaces can prevent larval feeding (see Fürstenberg‐Hägg et al., 2013 for a review), and trichomes can physically prevent larvae from accessing the leaf tissue, significantly hinder movement, exude harmful substances or may even cause integumental injuries (Despland, 2019; Fürstenberg‐Hägg et al., 2013; Gilbert, 1971). The evolution of toxins also helps plants to escape herbivory from many generalists (Birnbaum & Abbot, 2018; Wittstock & Gershenzon, 2002). Furthermore, toxic plants often also display more immediate responses to attack, such as the release of these concentrated toxins into sites of feeding damage (Denno & Benrey, 1997; Karban, 2011). These host plant defences, and the need to overcome them, are thought to be the main promoter of larval aggregation in some systems (Clark & Faeth, 1997; Denno & Benrey, 1997; Despland, 2019; Fordyce & Agrawal, 2001; Kawasaki et al., 2009; Rentería et al., 2022). For example, some larvae will meticulously remove leaf trichome tips to reduce their harmful impact (Cardoso, 2008), but this is likely to be a costly task for an individual. Some gregarious larvae are well‐equipped to deal with trichomes and collectively cover them in silk to avoid contact (e.g. Despland, 2019, 2021; Rathcke & Poole, 1975). Additionally, collective feeding is thought to benefit larvae that may be susceptible to their host's toxin‐release response if they can completely consume the leaf before it is flooded with toxins (Denno & Benrey, 1997).

Aside from their herbivores, ecological drivers from the immediate habitat are likely to shape host plant evolution, which in turn may influence lepidopteran evolution. For example, the spatial distribution, or density, of host plants might vary, affecting how easily females locate suitable oviposition sites. Females may therefore adjust their oviposition strategy in response to the relative difficulty of locating suitable hosts, laying most of their eggs once a suitable host is located if the chances of encountering another are low (Braby & Nishida, 2010). Egg clustering may naturally follow from females laying most of their eggs at one patch, with clumped eggs often giving rise to gregarious larvae (Clark & Faeth, 1998; Korb & Heinze, 2016).

Here, we use the Heliconiini butterfly tribe as a model system to study the influence of specific hostplant traits on the evolution of larval gregarious behaviour. All Heliconiini larvae feed on vines from the Passifloraceae family (de Castro et al., 2018), which offers a shared ecological context within which specific trait differences can be interrogated. Passifloraceae are highly diverse, varying widely in their overall growth morphology and defences against herbivory, such as egg‐mimicking structures to deter oviposition, extrafloral nectar rewards to attract predatory ants, and toxic chemical components in their tissues (de Castro et al., 2018). These chemicals form an important line of defence against herbivory from generalist species and, perhaps as a result, some Heliconiini have been driven into specialising on small numbers of hosts. Heliconiini larvae have evolved resistance to their host's toxins, often in correlation with increased specialisation (de Castro et al., 2021; Engler‐Chaouat & Gilbert, 2007; Merrill et al., 2013), and the ability to incorporate these toxins into their own chemical defences (Arias et al., 2016; de Castro et al., 2021; Engler‐Chaouat & Gilbert, 2007). Additionally, larval social behaviour varies across the Heliconiini, even between very closely related species, with repeated shifts to grouped egg‐laying and gregarious larvae (Beltran et al., 2007; McLellan et al., 2023). Although little is known about the behavioural mechanisms supporting these aggregations, at least some gregarious Heliconiini are trail followers (Pescador‐Rubio et al., 2011), suggesting these transitions reflect behavioural adaptations in larvae rather than simple variation in female egg laying. This variation, coupled with tribe‐wide estimates of the phylogenetic structure of the Heliconiini (Cicconardi et al., 2023; Kozak et al., 2015), positions these butterflies as a highly useful system with which to study behavioural evolution in response to host plant ecology.

By revisiting the evolution of larval gregariousness in Heliconiini, we take a phylogenetic comparative approach to identify where transitions to larval gregariousness have taken place across the phylogeny. Then, by exploring variation in host plant use between the two behavioural phenotypes, we test hypotheses regarding the host traits that shape the evolution of gregarious behaviour. Specifically, we test (i) whether gregarious larvae use a narrower range of host plant species than solitary larvae. (ii) Whether aggregated larvae are more likely to occur on hosts with larger leaves. (iii) Whether aggregated larvae are more likely to occur on hosts with better defences (here specifically leaf trichomes). And (iv) whether larval behaviour is associated with their host plant's habitat.

## 2 METHODS

### 2.1 Phylogenies and species lists

**FIGURE 1:**
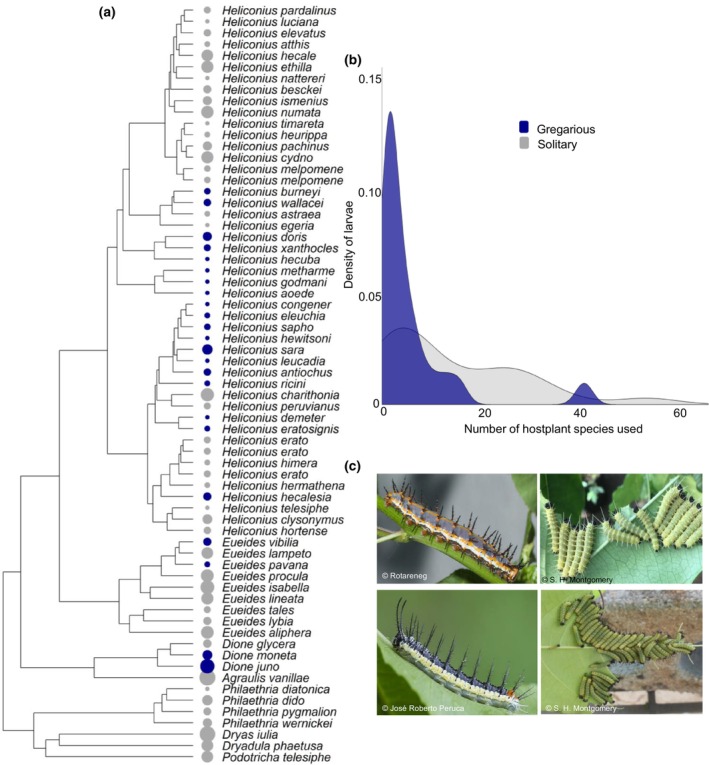
(a) The Heliconiini phylogeny, adapted from Kozak et al. (2015), showing larval social behaviour (solitary = grey tip points, gregarious = blue tips) and the number of separate Passifloraceae used by each species as larval host plants (1–57 represented by increasing tip point size). (b) The probability density of solitary (grey curve) and gregarious (blue curve) larvae which use each number of separate Passifloraceae host species. The majority of gregarious species use <20 hosts. (c) Image examples (with credit) of various Heliconiini larvae. Clockwise from top‐left: *Agraulis vanilla*, *Heliconius hewitsoni*, *Heliconius doris*, *Eueides isabella*.

We obtained two lists of 52 and 75 butterfly species within the Heliconiini tribe from phylogenies used in Cicconardi et al. (2023) and Kozak et al. (2015), respectively. Kozak et al.'s phylogeny was used for the primary analyses, as this incorporates a larger total number of species (Figure 1a). We also repeated all analyses using Cicconardi et al.'s phylogeny, as this is based on a greater amount of molecular data, and has some minor topological differences, but contains fewer species (see Appendix 1 for these results). Analyses using either phylogeny produced results that do not meaningfully differ.

### 2.2 Larval and host plant data

We collected data on larval ‘social behaviour’ (a trait including either solitary or gregarious character states) and Passifloraceae host species of the Heliconiini included in each phylogeny (all data with references can be viewed in online repository). Data on larval social behaviour, recorded as a binary variable (0 = solitary, 1 = gregarious) were based on previous classifications of egg clutch size range (Beltran et al., 2007; Brown Jr., 1981), with gregariousness recorded for any species with a range maximum ≥10 (as per Beltran et al.'s, 2007 categorisation). We note that larval aggregations may break down due to environmental conditions in some cases, but these occasions are likely to be rare and not affect our conclusions based on species‐wide data in a meaningful way. We additionally analysed social behaviour as a categorical variable [no aggregation (NA)/small aggregation (SA)/medium aggregation (MA)/large aggregation (LA)] as per records of four levels of increasing ‘sociability’ based on clutch size (Brown Jr., 1981): Solitary species (no aggregation) were recorded as zero. Small aggregated (SA) species were any with a median clutch size between 5 and 20, medium aggregated (MA) species were any with a median clutch size between >20 and 35 and large aggregated (LA) species were those with a median clutch size >35. Use of Passifloraceae host species was mainly taken from published sources (Benson et al., 1975; Beccaloni et al., 2008, as compiled and updated in Kozak, 2016 and Young et al., 2023). For a small number of species, all in the ‘Aoede’ (formerly ‘Neruda’) clade of the genus *Heliconius*, we only recorded the host at the genus level (*Dilkea*), as we lacked species‐level data, this meant we could not confirm how many separate species within this genus are used as hosts, despite there being 13 accepted species within *Dilkea* (POWO, 2022). The four species in the Aoede clade (three of which are included in our data) occur at lower densities than other *Heliconius* and lack some derived features of the genus. Re‐running the analyses with these genus‐level entries omitted had no meaningful effect on the results (Table A1).

Trait data on the Passifloraceae hosts were taken from two main sources (Benson et al., 1975; Ulmar & MacDougal, 2004). To determine whether larger leaves predict gregarious larvae, we recorded the mature leaf size of each Passifloraceae in our dataset, given by Ulmar and MacDougal as separate ranges (minimum and maximum) of leaf length and width (both cm). Here, we first calculated the median value of these ranges, then multiplied one median by the other to gain an estimate of the median leaf surface area, assuming an idealised leaf shape, which was used as a final value for what we term ‘leaf size’ (de Luna Souto et al., 2017). We acknowledge that, given the leaf shape diversity in *Passilfora*, the leaf surface area calculated here is likely a crude estimate of overall leaf tissue available to larvae. Leaf size data were heavily left‐skewed, and so were log transformed to normalise the distribution for analyses. To test if the presence of leaf trichomes predicts larval gregariousness, we recorded the vestiture (the presence or absence of leaf trichomes) of hosts as a binary variable (0 = glabrous (lacking trichomes), 1 = pubescent (possessing trichomes)), where leaves listed as ‘nearly glabrous’ were recorded as glabrous. Finally, to test for the effect of host habitat on larval behaviour, we recorded four distinct, main habitats (forest interior, forest edge, open areas and humid glades), taken exclusively from Benson et al. (1975).

### 2.3 Behavioural transitions

All analyses were performed using R v. 4.1.2 (R Core Team, 2013). We determined the phylogenetic signal of larval social behaviour, as estimated by Pagel's λ (Pagel, 1999), using the ‘fitDiscrete’ function in *geiger* (Pennell et al., 2014). The ‘fitDiscrete’ function estimates evolutionary transition rates between character states of traits with discrete levels, given available tip data, making it ideal for analysing our binary and categorical social behaviour trait. Additionally, ‘fitDiscrete’ allows for the comparison of models of evolutionary transition between solitary and gregarious states throughout the phylogeny. We used a likelihood ratio test to compare the null model, which assumes equal between‐state transition rates across the tree, against the all‐rates‐different model, which assumes the between‐state transition rate is different in either direction. We then used the ‘make.simmap’ function in *ape* (Paradis & Schliep, 2018), which simulates the character state evolution of a trait under a given model and allows for the exploration of discrete character evolution, making it ideal for our purposes. We used the best‐fitting transition rate model from our fitDiscrete analyses to estimate the behavioural character state at each node, and the number of independent transitions to gregariousness throughout the phylogeny. We constrained the root node to the solitary character state based on estimations from a larger butterfly phylogeny that the last common ancestor of Heliconiini was solitary with high (67%) confidence (McLellan et al., 2023). This improved confidence in estimations around basal nodes. When analysing categorical social behaviour, we used the ‘ace’ function in *ape*, which estimates the most likely character state at each node given available tip data. This function does not allow the option to constrain the root node, nor the use of the ‘all rates different’ evolution model with a multi‐levelled, discrete variable. We therefore performed the analyses under the ‘equal rates’ model. Additionally, we performed phylogenetic pathway analyses (PPA) using the package *phylopath* (von Hardenberg & Gonzalez‐Voyer, 2013) on categorical behavioural data and host plant usage data. PPA is used to test hypotheses against a null model (no relationship between traits) regarding the most likely order in which traits typically evolve. Our first model set exclusively tested social behavioural evolution, to test the hypothesis that increasing levels of gregariousness evolve from solitariness in a linear pattern. We included the number of larval host plants used in our second model set to investigate the order in which increased host specialisation and transitions to gregariousness typically evolve.

### 2.4 Tests of correlated evolution

We performed tests for correlated evolution between larval social behaviour and hostplant use, hostplant leaf size, hostplant vestiture and hostplant habitat, whilst controlling for phylogenetic effects, using MCMCglmm (Hadfield, 2010). The MCMCglmm package enables regression analyses between traits whilst accounting for potential trait value similarities between species due to relatedness by including the phylogeny as a random effect. We ran MCMC models for 5.1 million iterations, with a 0.1 million burn‐in and sample storage frequency of every 500 iterations, with significance of the model calculated as the probability of the parameter value being different from zero (*P*_MCMC_). We also report each model cofactor's posterior mean (*P*‐mean) and its 95% confidence intervals (CI). All analyses were performed using uninformative, parameter‐expanded priors for the random effect (G: *V* = 1, nu = 1, alpha.mu = 0, alpha.*V* = 1000; R: *V* = 1, nu = 0.002) and default priors for the fixed effects. Finally, the Heliconiini dataset on host plant use could potentially contain some uncertainties, as it combines records from geographically dispersed Heliconiini species. Hostplant use can vary across populations of single Heliconiini species (e.g. Merrill et al., 2013), potentially leading to overestimations in host use numbers for some species when data are combined over wide geographical ranges. We therefore also performed a more taxonomically limited analysis of a well‐studied community of 14 Heliconiini and nine *Passiflora* species in Panama (Merrill et al., 2013), as described above.

## 3 RESULTS

### 3.1 Many origins of gregarious larvae in Heliconiini

Overall, we recorded 23 gregarious species (Figure 1a), out of the 75 Heliconiini included in Kozak et al. (2015). Our model estimated there have been seven independent transitions to gregariousness across the Heliconiini phylogeny [under the ‘all rates different model’, model comparison: Χ^2^(1) = 4.812, *p* = .028, transition rate = 0.024]. Despite this pattern of convergent evolution, the phylogenetic signal of social behaviour is estimated to be very strong (λ = 1), likely reflecting the general tendency for transitions to occur at the base of specious clades. Transitions between solitary and gregarious states are most likely to occur at different rates, with 11 reversals from gregarious larvae back to solitary larvae across the phylogeny (transition rate = 0.078). This dynamic turnover of social behaviour suggests Heliconiini oviposition behaviour is likely responding to a range of selection pressures.

### 3.2 Species with gregarious larvae tend to be hostplant specialists, but there is no effect of leaf morphology

**FIGURE 2:**
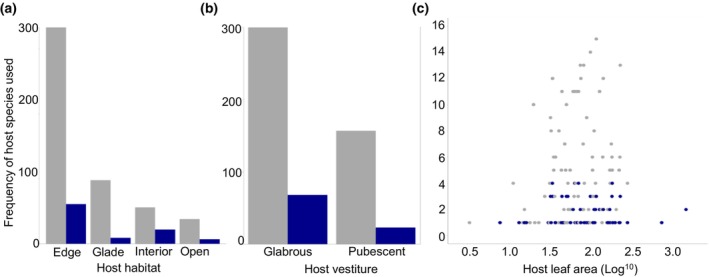
The frequencies at which Passifloraceae with certain traits are used by both solitary (grey bars and points) and gregarious (blue bars and points) Heliconiini larvae. (a) Larval use of host plants separated by their main habitat. (b) Larval use of hosts separated by their vestiture (presence or absence of leaf trichomes). (c) Larval use of host plants separated by their median mature leaf size.

Solitary larvae feed on a greater variety of host species than gregarious larvae (*P*‐mean = −0.920, 95% CI −1.622 to −0.235, *P*_MCMC_ = 0.015, Figure 1b). After omitting *Passiflora* hosts used by fewer than four larval species from the data, we found that *P. pedata*, which is host to five Heliconiini species, is more likely to be used by solitary larvae. Additionally, we found that more frequently used hosts by all larvae tend to grow in forest edge habitats (Table 1). Contrary to our predictions, we did not identify a relationship between specific host plant traits and larval social behaviour (Table 1; Figure 2).

**TABLE 1:** Two separate MCMCglmm sets testing the interactions between larval social behaviour, hostplant use frequency and hostplant traits using Heliconiini larvae included in Kozak et al.'s (2015) phylogeny.

Host trait	Levels	*P*‐mean	95% CI		*P*_MCMC_
			Lower	Upper	
Model set A					
Leaf size	N/A	2.113	−21.633	27.344	0.871
Vestiture	Glabrous/pubescent	−0.489	−17.046	13.641	0.914
Habitat	Edge	−92.001	−232.577	46.540	0.171
	Glade	−3.052	−43.149	28.011	0.815
	Interior	8.197	−22.514	43.876	0.614
	Open	−6.892	−49.978	28.816	0.793
Model set B					
Leaf size	N/A	−0.004	−0.311	0.297	0.983
Vestiture	Glabrous/pubescent	−0.015	−0.207	0.180	0.880
Habitat	Edge	2.741	0.878	4.451	0.004*
	Glade	0.039	−0.205	0.278	0.747
	Interior	−0.037	−0.315	0.235	0.806
	Open	0.008	−0.321	0.333	0.964

*Note*: Significant interactions are denoted by an asterisk. Model set A: larval social behaviour against host traits (*N* = 69). Model set B: larval hostplant use frequency against host traits (*N* = 67). Positive coefficients (*P*‐mean) indicate that host species with the given trait are used by many larvae, whereas negative coefficients indicate the host is less frequently used.

### 3.3 Gregariousness evolves semi‐linearly and is preceded by host specialisation

**FIGURE 3:**
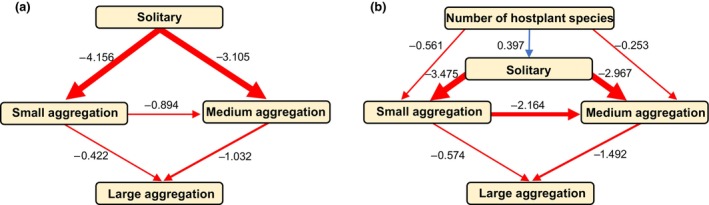
Results from phylogenetic pathway analyses (PPA) in which directional evolutionary relationships between traits are estimated. Arrows show the direction of evolutionary interactions, the values next to arrows display the pathway coefficient (strength of the estimated relationship). Between categories of social behaviour, negative coefficients (red arrows) indicate that a loss of the preceding trait occurs before a gain of the subsequent trait. (a) Output from the model looking only at larval social behaviour, showing support for the pathway in which small and medium aggregations occur before the evolution of large aggregations. (b) Output from the model including the degree of larval host specialisation (number of hostplant species used). With the number of hostplant species used as the preceding trait, the negative coefficients indicate that a decrease in the number of host species used (i.e. increased specialisation) precedes a transition to gregariousness.

Estimates from our PPA revealed that, from a solitary ancestor, small and medium aggregations are equally likely to evolve next (pathway coefficients: NA to SA = −4.156; NA to MA = −3.105). Our model also supports the pathway in which small aggregations evolve before medium aggregations (SA to MA = −0.894), and medium aggregations precede the evolution of large aggregations (MA to LA = −1.032, Figure 3a; Table A2). When host plant number is included in the model set, similar relationships between aggregation sizes as the first model are supported (Figure 3b). Our second model also suggests that a narrowing of larval diet breadth, with regards to hostplant species, precedes transitions to small and medium aggregations (host number to SA = −0.561; host number to MA = −0.253, Figure 3b; Table A2).

### 3.4 A focused assessment in a single community

Pooling data from multiple Heliconiini populations across a broad geographical range has the potential to skew host plant use data. Therefore, we repeated our analyses on a less species‐diverse, localised community of Heliconiini with an extensive history of ecological study. Using these data, we found no relationship between larval social behaviour and the number of different *Passiflora* species they feed on (*P*‐mean = −0.925, 95% CI −2.673 to 0.637, *P*_MCMC_ = 0.261). Whereas we support our finding of a lack of interaction between specific host plant traits and larval behaviour (Table A3), and we again observe that hosts which grow in forest edge habitats tend to be more frequently used by larvae (*P*‐mean = 0.931, 95% CI 0.225–1.621, *P*_MCMC_ = 0.027, Table A3).

## 4 DISCUSSION

Larval Lepidoptera have close evolutionary relationships with their host plants, where the phenotype of the latter often influences the traits of the former. We performed phylogenetic comparative analyses to explore this relationship, focusing on the evolution of Heliconiini larval behaviour in response to their Passifloraceae hosts. We find multiple origins and reversal of gregariousness across the Heliconiini phylogeny, with a tendency for related species to be similar in this trait. We also show that species with gregarious larvae tend to utilise a narrower range of hostplant species than those with solitary larvae, and that this specialisation likely occurs before the evolutionary transition to gregarious behaviour. The association between host plant specialisation and larval gregariousness identified here suggests that there are specific hostplant traits which select for the behaviour; however, we did not identify these traits here. We discuss our findings below.

Our findings support those of Beltran et al. (2007) in that there is likely to have been multiple independent evolutions to gregariousness across the Heliconiini. Larval gregariousness is mostly concentrated in distinct clades across the phylogeny (Figure 1a). This pattern is expressed quantitatively by the high phylogenetic signal of larval social behaviour. Additionally, our data reveal that larval gregariousness, measured by proxy of egg clutch size, increases over evolutionary time in a semi‐linear pattern, whereby transitions to small clutches from single eggs tend to precede much larger clutches (Figure 3a). This suggests that there may be physiological constraints to females laying large clusters of eggs, which are incrementally overcome by increasing smaller clutch sizes over evolutionary time, and/or a selection pressure feedback loop which promotes larger clutches once gregariousness evolves.

Our analyses of the relationship between Heliconiini larvae and their host plants support only one of our hypotheses, that gregarious species tend to be more specialised. Our data show that Heliconiini species with solitary larvae tend to feed on a greater variety of host species than gregarious larvae (Figure 1b), and that this increased host specialisation evolves before the transition to gregariousness. We offer two possible explanations for our observed association between larval gregariousness and host plant specialisation. First, species with gregarious larvae might specialise onto hosts with higher nutritional value, which are better positioned to support groups of larvae. This is supported by evidence that females of other butterfly species with gregarious larvae preferentially oviposit on higher‐quality hosts (Schäpers et al., 2016), although this preference may also be present in solitary species (Gripenberg et al., 2010). Second, maintaining specialism on toxic hosts, instead of switching to a non‐toxic host, may be a response to high inter‐specific competition for food resources (e.g. Merrill et al., 2013), as minimising additional, inter‐specific competition is likely to be disproportionately important to grouped larvae. The *Passiflora* have robust chemical defences, yet specialisation often allows larvae to minimise the fitness costs associated with metabolising their hosts' toxins, to a greater degree than achieved by closely related generalist species (e.g. de Castro et al., 2021; Engler‐Chaouat & Gilbert, 2007; Merrill et al., 2013). Furthermore, specialists are often better able at converting their host's toxins into their own defence, enhancing their toxicity, evidenced by the finding that both gregarious and specialist *Heliconius* species are more toxic than solitary and generalist species, respectively (Arias et al., 2016). Increased toxicity resulting from host specialisation might explain our finding that this behaviour evolves before gregariousness for most Heliconiini (Figure 3b), given that larval toxicity most likely precedes transitions to gregariousness (and aposematism before that) across the wider butterfly phylogeny (McLellan et al., 2023). We note that the findings from our focused assessment indicate that, at the local level, host plant specialisation does not significantly differ between solitary and gregarious Heliconiini, despite a similar interaction coefficient to the main model. This lack of significance is likely an effect of low power, indicated by the larger CI range in this second model, and the low numbers of hosts reportedly used by all species across this dataset.

Despite evidence of an effect of host plant diversity on gregarious behaviour, none of the specific host plant traits examined in this study act as predictors of Heliconiini larval gregariousness. Whereas host plant morphology is thought to influence the evolution of other larval traits, such as anti‐predator colour strategy (Prudic et al., 2007), our results do not show similar influences on social behaviour. First, our assumption was that leaves with larger surface areas would provide better ‘stages’ for larval aggregations. However, we found no effect of leaf size on larval social behaviour, suggesting that even small leaves may be big enough to support groups of larvae if larvae are small or the groups do not contain many individuals. We also predicted an evolutionary link between the presence of host plant leaf trichomes and larval gregariousness, such that aggregated feeding benefits may contribute towards a form of behavioural character displacement, where gregarious larvae specialise on well‐defended hosts that solitary species struggle to feed on. However, we found that the presence or absence of *Passiflora* leaf trichomes has no influence on larval social behaviour in the Heliconiini. This may be because of a lack of specificity in available vestiture data, meaning we could only record vestiture in binary format and lacked information on the length and density of trichomes on most of the pubescent plants. Other useful trichome data, particularly their structure (whether they are hooked, glandular or neither), are also absent from the literature. These features are likely to be important determinants of how difficult trichome defences are for larvae to overcome (Despland, 2019; Fürstenberg‐Hägg et al., 2013). Alternatively, it may simply be the case that, in most instances, aggregating does not improve larvae's ability to overcome trichomes to an extent that it is selected over solitary feeding.

Finally, we expected larval behaviour to vary according to the main habitat of their host plants (and by extension their own habitat), given the potential ecological differences between them. Whilst we are missing habitat data for a number of host species in this study, overall, we found no evidence that habitat predicts larval behaviour. Our inclusion of habitat type was based on the assumption that it may act as a proxy for ecological factors which potentially influence larval social behaviour, such as host spatial distribution (Braby & Nishida, 2010; Young, 1983). Our negative result may indicate that this assumption is not valid. However, in both our geographically broad and focused datasets, we observed that hosts growing on the edges of forest habitats are favoured by Heliconiini in general, regardless of social behaviour. This finding appears to support evidence of a preference for forest edge plants in various *Heliconius* species (e.g. Ramos & Freitas, 1999; Seixas et al., 2017). It is, therefore, possible that there is some aspect of this habitat, or the plants within it, that ovipositing females favour over others, although we cannot rule out a bias in how these data are recorded, such as edge habitats being easier to access than forest interiors.

In summary, larval gregariousness is moderately phylogenetically conserved and widespread across the Heliconiini, with multiple origins and reversals. However, the specific ecological drivers of larval gregariousness remain unclear. Variation in hostplant specialisation between solitary and gregarious Heliconiini larvae suggests that there are certain plant traits that promote aggregation; however, available data has not led to their identification. We did not include plant chemistry in our analyses, but this may have given us a better understanding of why larvae specialise onto certain hosts, for example, to escape competition from generalists. Currently, however, data on toxicity variation across populations of larvae and their hosts is lacking. It would also be of interest to explore how the greater levels of hostplant specialisation in gregarious species impact their population dynamics and spatial distribution, both locally and across their geographic range. Additionally, host spatial density, and whether it varies between habitats, is an important factor missing from our data. Focus on a single Heliconiini community, over multiple seasons, is needed to collect reliable data on the relative spatial distributions of Heliconiini hosts, which is a challenging endeavour. Nevertheless, we have identified that increased host specialisation frequently occurs before the evolution of gregarious behaviour in Heliconiini larvae. This suggests that there are key host plant traits which may predict this behaviour, which require further study to identify.

## AUTHOR CONTRIBUTIONS

**Callum F. McLellan:** Conceptualization (supporting); data curation (lead); formal analysis (lead); funding acquisition (equal); writing – original draft (lead); writing – review and editing (equal). **Stephen H. Montgomery:** Conceptualization (lead); formal analysis (supporting); funding acquisition (equal); supervision (lead); writing – review and editing (equal).

## FUNDING INFORMATION

The authors are funded by a Biotechnology & Biological Sciences UK (BBSRC) SWBio grant to C.F.M. BB/M009122/1 and Natural Environment Research Council UK (NERC) Fellowship NE/N014936/2 to S.H.M.

## CONFLICT OF INTEREST STATEMENT

The authors declare no competing interests.

## Data Availability

All data files and R scripts used in this study, along with citations to data sources, are available from Zenodo repository, DOI: 10.5281/zenodo.10264176. https://zenodo.org/records/10264176. Additional information is provided in the Appendix 1.

## APPENDIX 1

### APPENDIX METHODS

We collected data on larval social behaviour from Brown Jr. (1981) in binary, categorical and continuous formats. Brown provides the clutch size range of each Heliconiini species, which we used as a proxy for our categorical and continuous data on larval gregariousness. Categorical social behaviour was recorded as four levels (NA, SA, MA, LA), as explained in the main methods. We also used median clutch sizes for the continuous data and performed additional transition rate analyses on these categorical and continuous data.

**TABLE A1:** Results from MCMCglmms between larval social behaviour and hostplant traits using Kozak et al.'s (2015) phylogeny, where the host genus *Dilkea* has been omitted from the data (*N* = 66).

Trait	Levels	*P*‐mean	95% CI		*P*_MCMC_
			Lower	Upper	
Host use count	N/A	−0.783	−1.507	−0.078	0.035*
Leaf size	N/A	1.053	−18.640	19.467	0.879
Vestiture	Glabrous/pubescent	−1.248	−32.891	26.979	0.888
Habitat	Edge	−109.074	−338.546	42.085	0.163
	Glade	−10.849	−59.826	20.957	0.563
	Interior	5.811	−39.564	55.802	0.750
	Open	−15.566	−82.591	23.970	0.619

*Note*: Significant interactions are denoted by an asterisk.

**TABLE A2:** Pathway coefficients from the averaged best models in two separate PPA model sets, also given are the standard error (SE) and confidence intervals (CI) of each estimated pathway (social behaviour only set *N* = 69, with host number set *N* = 67).

Model set	Pathway	Path coefficient	SE	CI	
				Lower	Upper
Social behaviour only	NA → SA	−4.156	1.573	−7.238	−1.074*
	NA → MA	−3.105	1.428	−5.903	−0.307*
	SA → MA	−0.894	1.335	−3.510	1.722
	SA → LA	−0.422	1.067	−2.514	1.669
	MA → LA	−1.032	1.651	−4.269	2.205
Social behaviour and host number	HN → NA	0.397	0.308	−0.207	1.002
	HN → SA	−0.561	0.501	−1.543	0.421
	HN → MA	−0.253	0.450	−1.135	0.628
	NA → SA	−3.475	1.226	−5.878	−1.071*
	NA → MA	−2.967	1.169	−5.258	−0.675*
	SA → MA	−2.164	1.456	−5.017	0.689
	SA → LA	−0.574	1.099	−2.728	1.580
	MA → LA	−1.492	1.740	−4.903	1.918

*Note*: Traits are no aggregation (NA), three increasing sizes of aggregation (small/medium/large = SA/MA/LA) and the number of host plant species used by larvae (HN). CIs that do not include zero denoted with asterisk.

**TABLE A3:** Two separate MCMCglmm sets testing the interactions between larval social behaviour, hostplant use frequency and host plant traits using data from a community of 14 Heliconiini and nine *Passiflora* species in Panama (Merrill et al., 2013).

Host trait	Levels	*P*‐mean	95% CI		*P*_MCMC_
			Lower	Upper	
Model set A					
Leaf size	N/A	−247.7	−880.9	350.5	0.397
Vestiture	Glabrous/pubescent	−39.58	−205.41	93.53	0.665
Habitat	Edge	−109.00	−477.39	191.61	0.430
	Glade	51.98	−239.14	340.95	0.683
	Open	−628.03	−1454.66	180.88	0.118
Model B					
Habitat	Edge	0.931	0.225	1.621	0.027*
	Glade	0.059	−1.179	1.218	0.911
	Open	−1.462	−4.786	1.775	0.381

*Note*: Significant interactions are denoted by an asterisk. Model set A: larval social behaviour against host traits. Model B: larval host plant use frequency against host habitat. Positive coefficients (*P*‐mean) indicate that host species with the given main habitat are used by many larvae, whereas negative coefficients indicate the host is less frequently used.

Before testing for evolutionary relationships between larval social behaviour and various host plant traits, we performed a count on the host data to reveal how many different larval species use each plant as a host. Using these count data, we omitted hosts used by fewer than four separate larvae to create a more robust test for larval social behaviour against individual host species. We also used these count data to determine which hosts are used more frequently by larvae (Table A4).

**TABLE A4:** *Passiflora* host species that are used by the greatest number of Heliconiini larvae included in Kozak et al.'s (2015) phylogeny (*N* = 75).

Species	No. larvae used by	Growth habit	Habitat	Vestiture	Leaf area (cm)
*P. laurifolia*	21	Vine	Edge	Glabrous	51.75
*P. vitifolia*	18	Vine	Edge	Pubescent	115
*P. edulis*	16	Vine	Edge	Glabrous	225
*P. auriculata*	15	Vine	Edge	Glabrous	70
*P. oerstedii*	15	Vine	Edge	Glabrous	63
*P. biflora*	14	Vine	Glade	Glabrous	33.75
*P. nitida*	14	Vine	Edge	Glabrous	140
*P. capsularis*	13	Vine	Edge	Pubescent	60.13
*P. foetida*	13	Vine	Open	Pubescent	80.75
*P. suberosa*	13	Vine	Glade	Pubescent	74.79
*P. caerulea*	12	Vine	Edge	Glabrous	126
*P. coccinea*	12	Vine	Edge	Pubescent	50
*P. quadrangularis*	12	Vine	Edge	Pubescent	67.5
*P. quadriglandulosa*	12	Vine	Edge	Glabrous	32
*P. rhamnifolia*	12	Shrub	Interior	Pubescent	44
*P. sidaefolia*	12	Vine	Edge	NA	NA

*Note*: Included are the number of larval species which use each host and the host traits.

### APPENDIX RESULTS

#### Main analyses using an alternate phylogeny

We performed all our main analyses on an additional, separate Heliconiini phylogeny (Cicconardi et al., 2023). Overall, we recorded 19 gregarious species, out of 57 included in Cicconardi et al. (2023). Our model estimated eight independent transitions to gregariousness, and the phylogenetic signal of social behaviour is estimated to be very strong (λ = 1.0). Transitions between solitary and gregarious states are most likely to occur at equal rates (Χ^2^(1) = 2.146, *p* = .143, transition rate = 0.033), with four reversals from gregarious larvae back to solitary larvae across the phylogeny.

As reported in our main analysis, solitary larvae feed on a greater variety of host species than gregarious larvae (*P*‐mean = −0.855, 95% CI −1.673 to −0.083, *P*_MCMC_ = 0.039). After omitting *Passiflora* hosts used by fewer than four larval species from the data, we found no evidence of an interaction between larval social behaviour and any host species. Additionally, we found that more frequently used hosts by all larvae tend to grow in woods edge habitats (*P*‐mean = 2.989, 95% CI 1.322–4.681, *P*_MCMC_ = 0.001, Table A5). Similar to our main results, we did not identify any interactions between specific host plant traits, including leaf size, and larval social behaviour (Table A5).

**TABLE A5:** Two separate MCMCglmm sets testing the interactions between larval social behaviour, hostplant use frequency and host plant traits using Heliconiini larvae included in Cicconardi et al.'s (2023) phylogeny.

Host trait	Levels	*P*‐mean	95% CI		*P*_MCMC_
			Lower	Upper	
Model set A					
Leaf size	N/A	3.472	−16.374	27.147	0.796
Vestiture	Glabrous/pubescent	−6.667	−42.064	26.752	0.709
Habitat	Edge	−73.302	−225.487	83.437	0.293
	Glade	−6.971	−42.794	26.520	0.719
	Interior	3.366	−35.381	44.157	0.828
	Open	−7.981	−49.500	30.254	0.735
Model set B					
Leaf size	N/A	−0.014	−0.343	0.328	0.927
Vestiture	Glabrous/pubescent	−0.011	−0.227	0.197	0.930
Habitat	Edge	2.989	1.322	4.681	0.001*
	Glade	0.047	−0.204	0.326	0.727
	Interior	−0.043	−0.361	0.278	0.792
	Open	0.022	−0.348	0.391	0.907

*Note*: Significant interactions are denoted by an asterisk. Model set A: larval social behaviour against host traits (*N* = 55). Model set B: larval host plant use frequency against host traits (*N* = 53). Positive coefficients (*P*‐mean) indicate that host species with the given trait are used by many larvae, whereas negative coefficients indicate the host is less frequently used.

#### Transition rate analyses of categorical and continuous social behaviour data

When analysed as a categorical variable, we recorded nine SA, nine MA and five LA species out of 75 included in Kozak et al. (2015). The phylogenetic signal is strong (λ = 0.929) and not significantly different from 1.0 (Χ^2^(1) = 0.502, *p* = .478). Transitions between behavioural states are estimated to occur at different rates (Χ^2^(11) = 22.874, *p* = .018), with the highest transition rate estimated to be from MA to LA (transition rate = 0.188 vs. next highest SA to NA = 0.187).

When analysed as a continuous variable, larval social behaviour is estimated to follow an Ornstein–Uhlenbeck model of evolution (comparison with BM model: Χ^2^(1) = 8.800, *p* = .003), with a relatively weak mean reverting force (α = 0.104) towards the optimum clutch size (z0 = 11.290). Again, the phylogenetic signal is strong (λ = 0.862) and not significantly different from 1.0 (Χ^2^(1) = 1.587, *p* = .208).
